# Translational research on drug development and biomarker discovery for hepatocellular carcinoma

**DOI:** 10.1186/s12929-024-01011-y

**Published:** 2024-02-17

**Authors:** Valerie Chew, Chien-Huai Chuang, Chiun Hsu

**Affiliations:** 1 Translational Immunology Institute, SingHealth-DukeNUS Academic Medical Centre, Singapore, Singapore; 2 Duke-NUS Medical School, Singapore, Singapore; 3 Department of Medical Oncology, National Taiwan University Cancer Center, Taipei, Taiwan; 4 Graduate Institute of Oncology, National Taiwan University College of Medicine, Taipei, Taiwan; 5 Department of Oncology, National Taiwan University Hospital, Taipei, Taiwan

**Keywords:** Anti-angiogenic, Genomic, Immune checkpoint inhibitor, Multi-kinase inhibitor, Transcriptomic

## Abstract

Translational research plays a key role in drug development and biomarker discovery for hepatocellular carcinoma (HCC). However, unique challenges exist in this field because of the limited availability of human tumor samples from surgery, the lack of homogenous oncogenic driver mutations, and the paucity of adequate experimental models. In this review, we provide insights into these challenges and review recent advancements, with a particular focus on the two main agents currently used as mainstream therapies for HCC: anti-angiogenic agents and immunotherapy. First, we examine the pre-clinical and clinical studies to highlight the challenges of determining the optimal therapeutic combinations with biologically effective dosage for HCC. Second, we discuss biomarker studies focusing on anti-PD1/anti-PD-L1-based combination therapy. Finally, we discuss the progress made in our collective understanding of tumor immunology and in multi-omics analysis technology, which enhance our understanding of the mechanisms underlying immunotherapy, characterize different patient subgroups, and facilitate the development of novel combination approaches to improve treatment efficacy. In summary, this review provides a comprehensive overview of efforts in translational research aiming at advancing our understanding of and improving the treatment of HCC.

## Introduction

The development of new oncology drugs for unresectable HCC has been hindered by limited accessibility to early-phase clinical trials [1] as well as concerns of adverse events associated with chronic liver diseases or loco-regional therapy [2, 3]. Despite these challenges, the introduction of new treatment regimens, including multi-kinase inhibitors (MKIs), immune checkpoint inhibitors (ICI), and their combinations, has not only expanded the treatment options available for patients with advanced-stage HCC but also introduced new prospects of multi-modality treatments for patients with earlier stage diseases [4, 5].

Anti-PD1/ anti-PD-L1 ICI-based combination therapy is regarded as the most noteworthy breakthrough in systemic therapy for unresectable HCC. Although findings from recent, pivotal phase III randomized clinical trials play key roles in shaping the future development of novel systemic therapy [6–15], translational research aiming at elucidating antitumor mechanisms and characterizing patient subgroups who are most likely to benefit from specific treatments is also essential. In this review, we explore the progress of translational research on drug development for HCC treatment from three distinct perspectives, namely, the reader, the interpreter, and the creator, to illustrate how translational research may aid in advancing the understanding and improving the treatment of HCC. As outlined by Dr. Bijay Kumar Das, an Indian literature critic, “A translator is a reader, an interpreter and a creator all in one”. Researchers must be aware of the available literature on HCC treatment and drug development (as readers), capable of analyzing and understanding the complex clinical and pre-clinical data (as interpreters), and, ideally, able to use this knowledge and understanding to design new experiments, develop new drugs, and ultimately advance the field of HCC treatment (as creators).

In this review we focus on the aspects of drug development and biomarker discovery for the 2 major classes of agents that are currently the mainstream therapies for HCC, namely anti-angiogenic agents and ICIs. As readers, we examine how translational research is involved in the development of new drugs for HCC. We also reviewed the preclinical studies focusing on the immunomodulatory effects of anti-angiogenic agents for HCC, highlighting the potential benefits and challenges of using in vivo and in vitro models. As interpreters, we review the correlative biomarker studies from randomized trials of anti-PD1/ anti-PD-L1-based therapy and single-arm cohort studies. We also discuss the benefits and challenges of developing tissue- and blood-based predictive biomarkers and the confounding effects exerted by the underlying etiologies of liver diseases. As creators, we discussed recent advancements in the multi-omics analyses of the HCC micro-environment. Specifically, we focus on advancements in computational biology, which enhance our collective understanding of the complex interactions of immune cells in the tumor microenvironment (TME), and on the implications of these advancements for both efficacy and adverse events of immunotherapy. We also emphasize the need for developing novel pre-clinical models to support mechanistic exploration and biomarker identification. In summary, translational research is a complex and multifaceted process that requires researchers to be readers, interpreters, and creators.

### Readers: lessons learned from translational research on anti-angiogenic therapy for HCC

Traditionally, translational research aimed at developing new drugs for HCC has had 2 primary objectives: establishing reliable predictive biomarkers to develop tailored treatment options for specific patient populations and understanding the underlying mechanisms of the new drugs. Nevertheless, achieving these objectives has been challenging for HCC. Clinical diagnosis of HCC, based on clinical and imaging characteristics rather than histological proof, is standard for patients with established risk factors (cirrhosis, chronic viral hepatitis) [16]. In addition, phase III randomized trials of systemic therapy for unresectable HCC have often not required a histological diagnosis, thereby resulting in a lack of adequate tumor samples for correlative biomarker analysis. Moreover, almost all of the molecular aberrations found in HCC, which have been primarily identified in studies using tumor samples obtained from patients who underwent surgery, are not typical drivers of the carcinogenesis process and are undruggable through either monoclonal antibodies or small molecule inhibitors [17]. Although molecular classifications of HCC based on genetic or epigenetic features of the tumors have been proposed to predict clinical outcome, they may not help patient categorization for the development of specific targeted therapy [18, 19].

### Anti-angiogenesis: a plausible yet elusive drug target

Generally, current targeted agents used for HCC treatment primarily exert their anticancer effects through the inhibition of angiogenesis. Although extensive clinical and pre-clinical studies have been conducted, no reliable set of predictive biomarkers has been established for identifying patients who may benefit from antiangiogenic therapy. In addition, no reliable pharmacodynamic markers have been identified to monitor the extent of angiogenesis inhibition and to determine the correlation between anti-angiogenic effects and clinical efficacy. The difficulty of preclinical experimental models to recapitulate the clinical features of HCC emerging from an inflammatory or cirrhotic background further widens the gap between preclinical mechanistic research and clinical application [20, 21].

In terms of the development of the anti- vascular endothelial growth factor (anti-VEGF) antibodies, early studies of bevacizumab in murine models suggested that doses of 2.5 mg/kg twice weekly or higher may achieve adequate plasma concentrations and anti-angiogenic effects [22]. Multiple randomized phase 2 and 3 trials have examined the dose–response effects of bevacizumab, either as single-agent therapy or in combination with chemotherapy, in different types of cancer. In these trials, higher doses of bevacizumab were associated with a trend of better treatment benefit, in terms of superior objective response rate or survival, and higher risks of adverse events, including hypertension, proteinuria and vascular events [23–26]. Since most of the adverse events were generally well tolerated by the patients, a high dosage of bevacizumab (5.0 mg/kg/ week) was eventually used in almost all subsequent clinical trials to develop new combination regimens. Overall, these findings underscore the limitations of pre-clinical models for dose determination in real-world clinical trials.

Pharmacodynamic biomarkers for anti-angiogenic therapy, including functional imaging, immunohistochemistry, and levels of circulating cytokines or angiogenic progenitor cells, have been widely tested but none of them have achieved the reproducibility and robustness required as a companion diagnostic in clinical practice [27, 28]. For example, in developing the MKI regorafenib, which inhibits VEGF receptor (VEGFR), biomarker experiments, including DCE-MRI functional imaging and circulating VEGFR, indicated that daily regorafenib dosage of 120 mg or higher was necessary to elicit anti-angiogenic effects [29]. This finding laid the foundation for subsequent clinical trials on HCC and other types of cancer, leading to the current recommended dosage of 160 mg per day, 3-week on and 1-week off. However, this dosage was not well tolerated by most patients. A dose-escalation strategy for regorafenib, starting from 80 mg per day (half of the recommended dose of 160 mg per day), with incremental adjustments depending on patient tolerance until a median daily dosage of 100 mg to 120 mg was reached, has been proposed to achieve similar progression-free survival to that of patients who received the standard-dosage of regorafenib [30].

The aforementioned challenges are also present in the development of other anti-angiogenic strategies, such as in the modulation of pericyte function. Pericytes play a key role in the stabilization and maturation of vascular sprouts, a process that involves multiple signalling pathways, including the VEGF, platelet-derived growth factor, and angiopoietin/ Tie-2 pathways [31, 32]. Translational research platforms to characterize the interaction among multiple relevant mechanisms and to minimize the gaps between pre-clinical evidence and clinical efficacy/ safety are urgently need.

### Complex interaction between anti-angiogenetic agents and ICIs

Mechanistic exploration became much more complicated when researchers attempted to address the immune modulatory effects of anti-angiogenic therapy [33]. Pre-clinical studies revealed that VEGF-targeting therapy can activate antitumor immunity in many aspects, including increasing antigen presentation, activating effector T cells, and counteracting immune suppressor cells in the TME. In addition to VEGF-targeting, tumor angiogenesis can also be indirectly modulated by targeting various immune cells (e.g., tumor-associated macrophages, TAMs) or stromal cells (e.g., pericytes) in the TME. Specific targeting agents and epigenetic-modifying agents are under development to modulate these cells [34–36]. Given that many plausible targets are available, developing predictive biomarkers for patient selection and pharmacodynamic monitoring has become more challenging.

Hypoxia in the TME plays a key role in the immunomodulatory effects of anti-angiogenic agents. Although HCC is typically a hypervascular tumor, the high interstitial pressure resulting from its aberrant vasculature may paradoxically induce hypoxia and immune suppression in the TME [37, 38]. This hypoxia-induced immune suppression involves complex interactions among different immune cells, the stroma, and the cytokine network in the TME [39–42]. Therefore, to improve anti-tumor immunity, multiple agents targeting tumor-associated hypoxia have been studied [43, 44]. According to the theory of vascular normalization in anti-angiogenic therapy, using excessively high doses of anti-angiogenic agents may induce hypoxia, acidosis and immune suppression in the TME, whereas using low-doses of anti-angiogenic therapy may enhance antigen presentation and improve T cell trafficking and function [38]. Pre-clinical studies have also indicated that using lower doses of anti-angiogenic MKI may induce vascular normalization, reduce hypoxia, and improve antitumor immunity, whereas using higher doses of anti-angiogenic MKIs may paradoxically increase hypoxia and promote immune suppression [45].

Understanding the biologically effective dosage of targeted agents and their relevant antitumor mechanisms is essential for developing optimal anti-angiogenic regimens. In our pre-clinical studies on regorafenib, we used regorafenib at a dosage of 5 mg/kg/day in animal models to mimic the half daily recommended dose of regorafenib (i.e., 80 mg per day) in human, in accordance with the aforementioned pharmacokinetic study. We found that this low-dose of regorafenib was associated with enhanced interferon-gamma response, M1 macrophage polarization, and antitumor immunity, independent of its anti-angiogenic effects. Regorafenib inhibits the p38 kinase/ Creb1/Klf4 signaling pathway in macrophages, which may explain its macrophage-polarizing effects [46]. According to Shigeta et al. (2020), regorafenib at a dosage of 10 mg/kg/day in mouse liver cancer models may result in optimal vascular normalization and increased T-cell infiltration in the TME. Regorafenib may also increase the expression of CXCL10 by HCC cells and the intratumoral infiltration of CD8 + CXCR3 + T cells through the inhibition of STAT3 activity. These two mechanisms may account for the antitumor synergy observed between regorafenib and anti-PD1 therapy [47]. Overall, these studies have demonstrated how pre-clinical research can elucidate the optimal biologically effective dosage of targeted agents and their mechanisms of action.

In conclusion, the challenges and complexity in drug development and biomarker discovery are significant and must be addressed through reliable pre-clinical studies and solid mechanistic understanding. Overcoming these challenges ca aid in achieve actual progress in the clinical management of HCC, an unmet need that demands urgent attention.

### Interpreters: biomarker studies for the prediction of treatment efficacy and mechanistic exploration

Biomarkers are used clinically in risk stratification, early detection, diagnosis, prognosis, and treatment response prediction. Clinical parameters such as tumor size, tumor number, and liver functional reserves are incorporated in major HCC practice guidelines to recommend the choice of liver-directed therapy, such as chemo-embolization [4, 48–50]. For patients who require systemic therapy, no reliable set of biomarkers is yet validated for currently available treatment options. Treatment recommendations are typically based on the clinical and laboratory parameters defined in the pivotal clinical trials and on the safety concerns of specific agents and patient preferences [51].

Traditionally, biomarkers are developed per the principle of Occam’s razor, which posits that natural phenomena should be explained in the simplest form possible, with minimal assumptions [52]. This* i*s done to ensure test robustness, reduce intra- and inter-observer variations, and facilitate external validation in diverse patient populations [53]. The same principle is also used in the development of biomarkers for HCC. Currently the only predictive biomarker with level 1 evidence (proven by randomized trial (s) designed to test biomarker performance and clinical impact, according to the International Liver Cancer Association (ILCA) white paper [54] is alpha-fetoprotein (AFP) in selecting HCC patients for ramucirumab therapy (an anti-VEGFR antibody) in the second-line setting [55], although its usefulness is limited given the relatively low absolute survival gain by ramucirumab treatment.

**Fig. 1:**
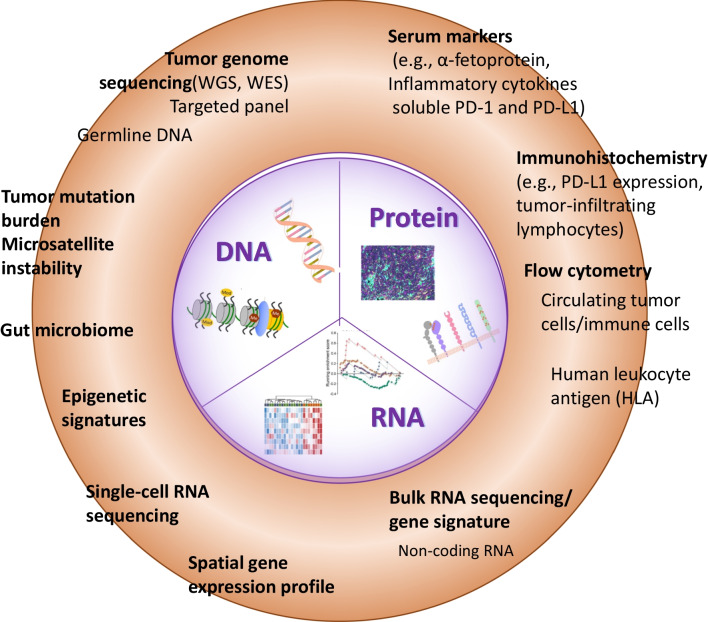
Approaches of biomarker development for immunotherapy in HCC. Summary of multi-omics profiling approaches of biomarker development. The biomarkers of DNA, RNA, and proteins are intergraded from different modalities. Each modality exhibits advantages and disadvantages for constructing the entire picture of tumors and microenvironments. WES, whole-exome sequencing, WGS, whole-genome sequencing

In ICI therapy, tumor PD-L1 expression and tumor mutation burden (TMB) are the most validated predictive biomarker for advanced cancers. More recently, multi-omics approaches are increasingly use to explore the mechanistic interaction among hosts, immune cells, and tumors for biomarker development (Fig. 1 and Table 1) [56]. Expression patterns or ‘signature’ of immune related genes in tumor tissue, particularly those related to inflammation and T cell function, may serve both for prediction of treatment efficacy and for mechanistic exploration [57, 58]. Biomarker studies using archival tumor tissues from HCC patients who received anti-PD1/ anti-PDL1 based therapy identified genes associated with inflammation, antigen presentation, interferon responses and cytokine signaling (ILCA level 2–3 evidence) [59–63]. However, findings from these translational studies cannot be easily validated externally because of difficulties in ensuring methodological standardization.

**Table 1:** Representative tissue- and blood-based biomarker results from clinical trials of ICI-based therapy for HCC

Bio-samples	Advantage/ disadvantage	Modality	Key findings
Tissue-based: • Tumour tissues RNA or DNA sequencing • Frozen or FFPE tumor samples	*Advantages*: Comprehensive multi-omics studies Opportunity to study spatial relationship of tumor and immune cells *Disadvantages*: Intra-tumoral heterogeneity Difficulty in collecting paired (before- and after-treatment) samples	Immune-related gene expression and signatures (RNA-seq)	• A 4-gene (PD-L1, *CD8A, LAG3,* and *STAT1*) signature associated with better response rate and survival (nivolumab; CheckMate 040) [59] • The Gajewski Inflammation signature and IL6-JAK-STAT3 signaling genes were associated with survival benefit (nivolumab; CheckMate 459) [60] • High effector T cell signature, regulatory T cell signature, and myeloid inflammation signature were associated better progression-free and overall survival (atezolizumab + bevacizumab; IMbrave 150) [63]
			• A 11-gene INFAP (interferon and antigen presentation) signature predicted response and survival in HCC patients treated with anti-PD-1 monotherapy [61] • A 9-gene exhausted CD8 T-cell signature expression associated with response to ICI therapy. [62]
		Whole-exome NGS Target panel NGS	• No consistent association between tumor mutation burden and response or survival [60, 63, 75] • Wild-type CTNNB1 or TERT promoter mutation associate with improved survival [63] • Activating alteration WNT/β-catenin signaling associate with lower response and shorter survival benefits [75]
		Epigenetic signature	• Epigenetic regulators (EGRs) score could predict clinical outcome in HCC patients treated with immunotherapy [80] • N6-methyladenosine (m6A) modification-related epigenetic signature as biomarker for response to anti-PD-1 immunotherapy in patients with HCC [81]
		Immunohistochemical staining	• Trend of higher objective response rate in tumors with increased PD-L1 expression (nivolumab, atezolizumab + bevacizumab) [59, 63] • Trend of higher objective response rate and better survival in tumors with increased PD-1 + cells (nivolumab) [59] • Tumor cell PD-L1 expression of 1% and greater and less than 1%: no differences in medial overall survival (nivolumab; CheckMate 459) [12] • Increased CD3 and CD8 showed a non-significant trend towards improved OS, and macrophage markers (CD68, CD163) were not associated with OS. [59]
		Multiplex IHC staining	• Higher density of infiltrating CD8 + T cells, CD3 + T cells, GZMB + CD3 + T cells in tumors, and MHC class I protein in responders [63] • Higher density of CD8 + LAG3 + cells in the tumors by multiplex immunofluorescence staining in responders (ICI) [74]
Blood-based: • PBMCs • Serum proteins (e.g., cytokines) • Ct- or cf-DNA • Epigenetic signature	*Advantages*: non-invasive; real-time monitoring *Disadvantages*: Technical challenges of detailed phenotypical characterization Challenge to link the mechanisms to the local tumor microenvironment	Flow cytometry	• Increase in CD8 + Ki67 + T cells early after treatment associated with higher objective response rate (durvalumab + tremelimumab) [94] • Higher baseline level of PD-1 + CD4 + T cells in patients who responded to the therapy [83] • Lower posttreatment NLR and PLR ratios are associated to better response in nivolumab treated HCC patients [60]
		CyTOF and scRNA seq	• Increased CXCR3 + CD8 TEM and APCs are associated to better response to anti-PD-1 ICI treatment [93]
		Serum AFP test	• Lower serum AFP of < 400 ng/mL was associated with superior OS in Nivolumab treated HCC patients (Checkmate040) [59] • AFP cutoffs of ≥ 75% decrease and ≤ 10% increase from baseline at 6 weeks were associated with longer OS and PFS [50] • Low AFP (< 100 ng/mL) and low C-reactive protein (< 1 mg/dL) were associated with better survival and treatment outcome (the CRAFITY score) [90–92]
		ctDNA or cfDNA sequencing	• Circulating WNT pathway-related mutations were not associated with clinical outcomes in immunotherapy treatment patients [89] • TERT ctDNA mutation predicts shorter OS in HCC patients treated with Atezo/Bev therapy [63]

HCC, hepatocellular carcinoma; FFPE, formalin-fixed, paraffin-embedded; PBMCs, peripheral blood mononuclear cells; ctDNA, circulating tumor DNA; cfDNA, cell-free DNA; NGS, next-generation sequencing; CyTOF, mass cytometry by time of flight; scRNA seq, single-cell RNA sequencing; AFP, alpha-fetoprotein; NLR, neutrophil–lymphocyte ratio; PLR, platelet–lymphocyte ratio; TEM, effector memory T cells; APCs, antigen-presenting cells; OS, overall survival

The role of epigenetic aberrations, including non‐coding RNA expression, DNA promoter hypo‐ or hyper‐methylation, and histone modifications (e.g., acetylation), in hepatocarcinogenesis and their potential as prognostic or predictive biomarkers have been extensively studied [64]. Epigenetic aberrations not only contribute to carcinogenesis but also are involved in TME remodeling, immune evasion, and effector T cell exhaustion [65–67]. Reversing epigenetic aberrations using de-methylating agents, histone deacetylase (HDAC) inhibitors, or enhancer of zeste homologue 2 (EZH2) inhibitors, may increase the efficacy of ICI therapy [68–70]. Therefore, developing epigenetic biomarkers and targets for immune modulation is another promising approach for enhancing ICI-based combination therapy.

### Tissue-based biomarker exploration

Tumor PD-L1 expression is associated with a favorable objective response to anti-PD1/ anti-PD-L1 therapy in both the preclinical models of liver cancer [71] and in clinical trials on patients with HCC [12, 59, 63]. As shown in Table 1, higher density of infiltrating T cells, particularly CD8 + T cells, CD3 + T cells, GZMB + CD3 + T cells, as well as MHC class I protein expression were observed in patients responding to combination immunotherapy with atezolizumab and bevacizumab [63]. Because HCC is associated with a lower TMB compared with other types of cancer, TMB is not useful for predicting immunotherapeutic response in HCC [60, 72]. Another likely explanation for this phenomenon is the high intra-tumoral heterogeneity of HCC, which makes obtaining an accurate measurement of the TMB from a single biopsy sample difficult [73, 74]. These findings clearly underscore the limitations of the minimalist approach for accurately predicting the therapeutic response in highly heterogeneous types of cancer such as HCC. Activation of the WNT/ β-catenin pathway was associated with inferior treatment efficacy in some [75, 76] but not all [63] studies of patients who received anti-PD1 therapy.

Overall, the composition of genes used to represent specific immune related pathways have varied from one study to another, rendering cross comparisons difficult. For instance, in the CheckMate-040 study of nivolumab, 4 genes, namely *CD274* (PD-L1), *CD8A, LAG3,* and *STAT1*, were selected to constitute an inflammation-related gene signature [59]. By contrast, in the CheckMate459 trial of nivolumab versus sorafenib, the Gajewski inflammation signature [77] was used to identify patients with better objective response and survival after nivolumab therapy [60].

The biomarker study for the atezolizumab plus bevacizumab combination therapy integrated data from the IMbrave150 randomized trial (atezolizumab plus bevacizumab versus sorafenib) and an earlier phase I trial to explore predictors of efficacy of the combination treatment and the synergistic immune modulatory mechanisms of anti-VEGF agent [63]. The team found that a higher objective response rate and longer survival were associated with higher PD-L1 expression, stronger effector T cell signatures (*CXCL9*, *PRF1*, and *GZMB*), and lower expression of certain metabolism-related pathways (e.g., bile acid, fatty acid). The additional therapeutic benefit of bevacizumab was associated with increased expression of genes related to regulatory T (Treg) cells (*CCR8*, *BATF*, *CTSC*, *TNFRSF4*, *FOXP3*, *TNFRSF18*, *IKZF2*, and *IL2RA)* and myeloid inflammation *(CXCL1*, *CXCL2*, *CXCL3*, *CXCL8*, *IL6*, *PTGS1*). Consistently, elevated expressions of effector T cells and myeloid inflammation signatures have been correlated with improved efficacy of atezolizumab plus bevacizumab for patients with advanced renal cell carcinoma [78].

The aforementioned findings support the potential use of transcriptomic markers for investigating the mechanisms of ICI-based combination therapy across different types of cancer. Recent advancements in epigenetic signatures have propelled multi-omics analysis into a new frontier [79, 80]. Some studies on HCC have established links between epigenetic-related gene signature (extracted from bulk RNA sequencing data) and immunotherapeutic responses [81, 82]. Although these studies have provided insights into the complex interactions between different immune cells in regulating antitumor immunity in the TME, a dauntingly high level of analytic expertise is required. In addition, classifying patients into subgroups of high- versus low-expression of specific signatures, based usually on median expression values of the particular patient cohorts, may hinder external validation in different patient cohorts.

### Blood-based biomarker exploration

Blood-based biomarker analysis enables non-invasive, real-time monitoring of treatment effects. In patients who received ICI-based therapy, real-time monitoring may aid in the development of pharmacodynamic markers to characterize immune activation after treatment [83] and differentiate between true and pseudo-progression after treatment [84]. Biomarkers detected in patients’ blood may reflect the tumor burden [85, 86], status of systemic inflammation (e.g., neutrophil–lymphocyte ratio (NLR) and platelet–lymphocyte ratio (PLR)), and the genetic features associated with specific biological behaviors [59, 87–89]. NLR and AFP levels were reported to predict response to ICI-based therapy [59, 85]. The CRAFITY score, consisting of serum C-reactive protein and AFP levels [90], may serve as both a prognostic factor and a predictor of efficacy for ICI-based systemic therapy for patients with advanced HCC [91, 92]. These data offer level 2 evidence, in accordance with ILCA guidelines, regarding the use of blood-based biomarkers in patients with advanced HCC. Circulating immune cells, particularly CD8 effector memory T cells and antigen presenting cells (APCs) are linked to objective immunotherapeutic response [93].

In addition to circulating immune cells, circulating tumor DNA (ctDNA) and cell-free DNA (cfDNA) are regarded as potential peripheral biomarkers for predicting immunotherapeutic response (Table 1). In patients with advanced HCC who received ICI-based therapy, high ctDNA levels and TERT mutations detected in ctDNA are associated with poor survival outcome [88, 89]. However, these biomarkers exhibit complex interactions with each other, and correlation with the same markers at tissue level needs further clarification. For example, genetic studies of the IMbrave150 trial indicated that patients with TERT promoter mutation in tumors are more likely to benefit from the combination therapy [63], but in another case series HCC patients with TERT mutation detected in circulatory DNA had inferior survival compared with patients without detectable TERT mutation [89].

The pharmacodynamic monitoring of ICI-based therapy may include analysis of T-cell activation or exhaustion by flow cytometry and T-cell clonality by T-cell receptor sequencing [83]. A dose optimization study of durvalumab (anti-PDL1) plus tremelimumab (anti-CTLA4) for patients with non-small-cell lung cancer revealed an increasing trend of T cell proliferation and activation in peripheral blood with increasing tremelimumab dosage. The dosage of 1 mg/kg was finally selected based on safety data [94]. In another similar study on patients with advanced HCC, tremelimumab (300 mg, single-dose) plus durvalumab induced a significant increase in the number of CD8 + Ki67 + T cells, informing the optimal dosage for the HIMALAYA trial [95]. These pharmacodynamic markers may oversimplify the immune regulatory effects of anti_CTLA4 agents. Preclinical studies suggest that the antitumor efficacy of anti-CTLA4 ICIs involves the inhibition of Treg cells [96, 97]. Therefore, more sophisticated technology are required to capture the complex interaction among immune cells in the TME. High dimensional immune-monitoring technologies such as mass cytometry by time-of-flight (CyTOF) combined with single-cell RNA sequencing (scRNA seq) can be used to identify specific immune subsets related to response and immune-related adverse events (irAEs), indicating the possibility of targeting novel immune regulatory pathways to uncouple treatment efficacy and irAEs [93].

### Confounders in the interpretation of biomarker studies

The etiologies of the underlying liver diseases have been extensively studied as confounding factors for the interpretation of clinical trial results. Meta-analyses of clinical trials on sorafenib indicated that patients with HCC and hepatitis C infection may benefit more from sorafenib treatment [98, 99]. Although molecular pathogenesis studies have suggested that HCC with different etiologies is associated with different patterns of molecular aberrations, these aberrations are not directly related to the antitumor mechanisms of sorafenib and may not explain the difference in survival benefit among different sub-groups [100]. For HCC patients with different etiologies, no evident difference in survival benefit was noted for other targeted therapeutic regimens, including lenvatinib, regorafenib, cabozantinib, and ramucirumab [55, 101–103].

In ICI therapy, etiology-related debates have focused on non-viral etiologies, particularly non-alcoholic steatohepatitis (NASH) [104]. Pre-clinical models indicated that diet-induced NASH may compromise T cell function in the liver microenvironment and confer resistance to anti-PD1 therapy [104–106]. In response to metabolic stimuli, a subgroup of CXCR6 + CD8 T cells was identified to induce liver damage (‘auto-aggressive’) and may induce resistance to antiPD1/ antiPD-L1 therapy [104, 106]. A meta-analysis of ICI-based systemic therapy for advanced HCC suggested that patients with hepatitis B (HBV)-related HCC demonstrated more prominent survival benefit, whereas patients with non-viral HCC appeared to benefit the least (Table 2) [104].

**Table 2:** Comparison of overall survival benefits among patients with different etiologies of HCC who received anti-PD-1/ anti-PDL1 based therapy

Etiology	Study	OS hazard ratio (95% CI)	No of subjects ICI-based therapy/Control	
Non-viral	IMbrave 150	0.91 (0.52–1.59)	100	53
	KEYNOTE-240	0.88 (0.64–1.21)	163	85
	CheckMate-459	0.95 (0.74–1.22)	168	168
	RATIONALE-301	0.78 (0.55–1.12)	82	80
	COSMIC-312	1.18 (0.78–1.79)	169	86
	LEAP-002	0.86 (0.66–1.13)	118	133
	Camrelizumab + rivoceranib, 2022	0.65 (0.36–1.20)	42	45
	HIMALAYA	0.74 (0.67–0.95)	161	166
HCV-HCC	IMbrave 150	0.43 (0.21–0.87)	72	36
	KEYNOTE-240	0.96 (0.48–1.92)	43	21
	CheckMate-459	0.71 (0.49–1.01)	87	86
	RATIONALE-301	0.64 (0.38–1.08)	46	39
	COSMIC-312	1.10 (0.72–1.68)	136	67
	LEAP-002	0.86 (0.60–1.24)	94	87
	Camrelizumab + rivoceranib, 2022	0.56 (0.22–1.45)	22	29
	HIMALAYA	1.06 (0.76–1.49)	110	104
HBV-HCC	IMbrave 150	0.51 (0.32–0.81)	164	76
	KEYNOTE-240	0.57 (0.35–0.93)	72	29
	CheckMate-459	0.77 (0.56–1.05)	116	117
	RATIONALE-301	0.91 (0.73–1.14)	214	213
	COSMIC-312	0.53 (0.33–0.87)	127	64
	LEAP-002	0.75 (0.58–0.97)	192	193
	Camrelizumab + rivoceranib, 2022	0.53 (0.41–0.68)	208	197
	HIMALAYA	0.64 (0.48–0.86)	122	119

However, the difference in survival benefit among HCC patients with viral versus non-viral etiologies was not consistently seen [9, 14, 107, 108]. The non-viral subgroups included in HCC clinical trials encompass a heterogeneous population of patients with different etiologies or underlying liver diseases which are usually less stringently diagnosed on the basis of current clinical practice guidelines [109]. In addition, the co-existence of metabolic dysfunction-associated fatty/steatotic liver disease (MAFLD or MASLD) is often overlooked in these trials [110–112]. MAFLD may coexist in about 10–20% patients with chronic viral hepatitis. It may also exacerbate liver inflammation and fibrosis, leading to poorer clinical outcomes than those of patients without MAFLD [111–114]. Therefore, it is reasonable to hypothesize that the presence of MAFLD with chronic viral hepatitis may modulate the immune microenvironment of HCC and complicate the interpretation of biomarkers for immunotherapy.

In addition to the underlying liver diseases, tumor-related features such as hypoxia and epithelial-mesenchymal transitions (EMT) also play important role in determining treatment responses. In a previous study we described the effect of hypoxia on the enrichment of and interaction between immunosuppressive dendritic cells (DCs) and Treg [39]. Hypoxia may serve as a confounding factor that further attenuates immunotherapeutic responses because of its immunosuppressive effect. Consistent with our findings, those of Kopecka et al. [115] suggested hypoxia is a potential driver of resistance to immunotherapy. EMT is associated with tumor immune escape [116], which may regulate the expression of immune checkpoint molecules [117]. Further research is required to determine the impact of this phenomenon on HCC immunotherapy.

In summary, development of an improved technology or system is required to address the limitations of current biomarkers and the potential confounding effects from underlying etiologies and tumor characteristics.

### Creators: advancement in multi-omics approach for translational research in HCC

In published clinical trials of anti-PD1/ anti-PD-L1-based combination therapy for unresectable HCC, efficacy appears to a plateau, with an overall survival of 20 months, a progression-free survival of 7–8 months, and an objective tumor response approximately 25% based on RECIST 1.1 (response evaluation criteria in solid tumors, Table 3) [48]. Several approaches can be considered to enhance the efficacy of systemic therapy. The first approach is combination with agents targeting other immune checkpoints, such as TIGIT (T Cell Immunoreceptor with Ig and ITIM Domains), to enhance T-cell function [118]. The second approach is targeting mechanisms of resistance to anti-PD1/ anti-PD-L1 therapy identified in pre-clinical research, such as tumor-infiltrating Treg cells [39, 119], epigenetic control of immune function [120, 121], and other immune-related signaling pathways [122]. The third approach is exploring novel targets for immune modulation. Further research is required to comprehensively understand the phenotypes and functions of various immune cell subsets within the TME. In recent years, various multi-omics approaches, particularly the single-cell omics (SC-omics) technologies, have been developed, providing a more in-depth understanding of the heterogenous and complex dynamics between different sub-populations within the TME.

**Table 3:** Representative clinical trials of ICI-based systemic therapy for unresectable HCC

Study	Mechanism of action	Treatment (no. of subjects)	Overall survival (OS)/ Progression-free survival (PFS) (months) (median/ 95% C.I.)	Hazard ratios	Objective response rate (%, 95% C.I.) RECIST 1.1/ modified REICST
ICI-based combination					
Finn, 2020; Cheng 2021 (IMBrave150)	Anti-PDL1 plus anti-VEGF	Atezolizumab 1200 mg + bevacizumab 15 mg/kg Q3W (336)	19.2 (17.0–23.7)/ 6.9 (5.7–8.6)	OS: 0.66 (0.52–0.85) p < 0.001#	30.0 (25.0–35.0)/ 33.2 (28.1–38.6)
	Multikinase inhibitor (MKI)	Sorafenib 400 mg BID (165)	13.4 (11.4–16.9)/ 4.3 (4.0–5.6)	PFS: 0.65 (0.53–0.81) p < 0.001#	11.0 (7.0–17.0)/ 13.3 (8.4–19.6)
Ren, 2021 (ORIENT-32)	Anti-PD1 plus anti-VEGF	Sintilimab 200 mg + bevacizumab biosimilar 15 mg/kg Q3W (380)	Not reached/ 4.6 (4.1–5.7)	OS: 0.57, (0.43–0.75) p < 0.0001#	21 (17–25)/ 24 (20–29)
	MKI	Sorafenib 400 mg BID (191)	10.4 (8.5–NE) / 2.8 (2.7–7.0)	PFS: 0.56, 9 (0.46–0.70) p < 0.0001#	4 (2–8)/ 8 (4–13)
Kelley, 2022 (COSMIC-312)	Anti-PDL1 plus MKI	Atezolizumab 1,200 mg Q3W + cabozantinib 40 mg QD (432)	15.4 (13.7–17.7)/ 6.8 (5.6–8.3)	OS: 0.90 (0.69–1.18) p = 0.44	11.0 (8.1–14.2)/ NA
	MKI	Sorafenib 400 mg BID (217)	15.5 (12.1–NE)/ 4.2 (2.8–3.2)	PFS: 0.63, (0.44–0.91) p = 0.0012#	4.0 (1.6–7.1)/ NA
Abou-Alfa, 2022 (HIMALAYA)	Anti-PDL1 plus anti-CTLA4	Durvalumab 1500 mg Q4W + Tremelimumab 300 mg 1 dose (393)	16.4 (14.2–19.6)/ 3.78 (3.68–5.32)	OS: 0.78 (0.65–0.93) p = 0.0035#	20.1/ NA
	MKI	Sorafenib 400 mg BID (389)	13.8 (12.3–16.1)/ 3.6 (3.2–3.8)	PFS: 0.90, (0.77–1.05) p = NS	5.1/ NA
Finn, 2022 (LEAP-002)	Anti-PD1 plus MKI	Pembrolizumab 200 mg Q3W + Lenvatinib 8 mg (BW < 60 kg) or 12 mg (BW ≥ 60 kg) QD (395)	21.2 (19.0–23.6)/ 8.2 (6.4- 8.4)	OS: 0.84, (0.70–0.99) p = 0.0227	26.1/ 40.8
	MKI	Lenvatinib 8 mg (BW < 60 kg) or 12 mg (BW ≥ 60 kg) QD (399)	19.0 (17.2–21.7)/ 8.0 (6.3–8.2)	PFS: 0.83, (0.71–0.97) p = 0.0466	17.5/ 34.1
Qin, 2022	Anti-PD1 plus MKI	Camrelizumab (200 mg Q2W + rivoceranib 250 mg QD (272)	22.1 (19.1–27.2)/ 5.6 (5.5- 6.3)	OS: 0.62, (0.49–0.80) p < 0.0001#	25.4 (20.3–31.0)/ 33.1 (27.5–39.0)
	MKI	Sorafenib 400 mg BID (271)	15.2 (13.0–18.5)/ 3.7 (2.7–3.7)	PFS: 0.52, (0.41–0.65) p < 0.0001#	5.9 (3.4–9.4)/ 10.0 (6.7–10.2)
Single-agent ICI					
Finn, 2020 (KEYNOTE-240)	Anti-PD1	Pembrolizumab 200 mg Q3W (278)	13.9 (11.6–16.0)/ 3.0 (2.8–4.1)	OS: 0.78, (0.61–0.99) p = 0.0238	18.3 (14.0–23.4)/NA
	Placebo	Placebo (135)	10.6 (8.3–13.5)/ 2.8 (1.6–3.0)	PFS: 0.71, (0.57–0.90) p = 0.0022	4.4 (1.6–9.4)/NA
Yau, 2022 (CheckMate-459)	Anti-PD1	Nivolumab 240 mg Q2W (371)	16.4 (13.9–18.4)/ 3.7 (3.1–3.9)	OS: 0.85, (0.72–1.02) p = 0.075	15 (12–19)/NA
	MKI	Sorafenib 400 mg BID (372)	14.7 (11.9–17.2)/ 3.8 (3.7–4.5)	PFS: 0.93, (0.79–1.10) p = NS	7 (5–10)/NA
Abou-Alfa, 2022 (HIMALAYA)	Anti-PD1	Durvalumab 1500 mg Q4W (393)	16.4 (14.2–19.6)/ 3.8 (3.7–5.3)	OS: 0.78, (0.65–0.93) p = 0.0674 noninferiority	17.0/NA
	MKI	Sorafenib 400 mg BID (389)	13.8 (12.2–16.1)/ 4.1 (3.8–5.5)	PFS 0.90, (0.77–1.05) p = NS	5.1/NA
Qin, 2022 (Rationale-301)	Anti-PDL1	Tislelizumab 200 mg Q3W (342)	15.9 (13.2–19.7)/ 2.1 (2.1–3.5)	OS: 0.85, (0.71–1.01) p = 0.0398 noninferiority	14.3 (10.8–18.5)/NA
	MKI	Sorafenib 400 mg BID (332)	14.1 (12.6–17.4)/ 3.4 (2.2–4.1)	PFS: 1.11, (0.92–1.33) p = NS	5.4 (3.2–8.4)/NA
Qin, 2022 (KEYNOTE-394)	Anti-PD1	Pembrolizumab 200 mg Q3W (300)	15.9 (13.2–19.7)/ 2.1 (2.1–3.5)	OS: 0.79, (0.63–0.99) p = 0.0180#	12.7 (9.1–17.0)/ NA
	Placebo	Placebo (153)	13.0 (10.5–15.1)/ 2.3 (1.4–2.8)	PFS: 0.74, (0.60–0.92 p = 0.0032#	1.3 (0.2–4.6)/ NA

^#^: statistically significant difference as defined by the trial protocol

^1^RECIST: the types of response a patient can have been a complete response (CR), a partial response (PR), progressive disease (PD), and stable disease (SD). CR: Disappearance of all target lesions. PR: At least a 30% decrease in the sum of diameters of target lesions, taking as reference the baseline sum diameters. PD: At least a 20% increase in the sum of diameters of target lesions, taking as reference the smallest sum on study. Stable Disease (SD): Neither sufficient shrinkage to qualify for PR nor sufficient increase to qualify for PD, taking as reference the smallest sum diameters while on study

^2^Modified RECIST: estimating the reduction in “viable tumor volume” for HCC, using the same definition of volume change as RECISIT

^3^Objective response rate: CR + PR proportion

^4^Overall survival (OS) is defined as the time from randomization to death; progression-free survival (PFS) is defined as the time from randomization to progression or death. Imaging response assessment will be done according to study protocol and the RECIST.

^5^QD: once daily; BID: twice daily; Q2W: every 2 weeks; Q3W: every 3 weeks; Q4W: every 4 weeks; NA: not available

SC-omics technologies enable high-throughput profiling of individual cells and play a key role in elucidating the complex interplay between different immune subsets within the TME. These technologies, which encompass proteomics transcriptomics, genomics and even epigenomics, offer valuable insights into the immune evasion mechanisms used by cancer cells and potential targets for immunotherapy by identifying distinct immune cell populations and their associated functional states. Recent developments in single-cell epigenomics analysis, such as in single-cell transposase-accessible chromatin with sequencing [123] and spatial transcriptomics [124], have revolutionized our understanding of the TME and its response to immunotherapy. SC-omics approaches are particularly useful in identifying rare cell types, capturing transcriptional heterogeneity within cell populations, and unveiling the dynamic changes in cell states over time. Integrating SC-omics data with other omics approaches can provide a more comprehensive understanding of the molecular mechanisms driving cancer development and progression, thereby guiding the development of personalized cancer therapies.

### Multi-omics approaches for understanding the immune mechanisms in HCC

**Fig. 2:**
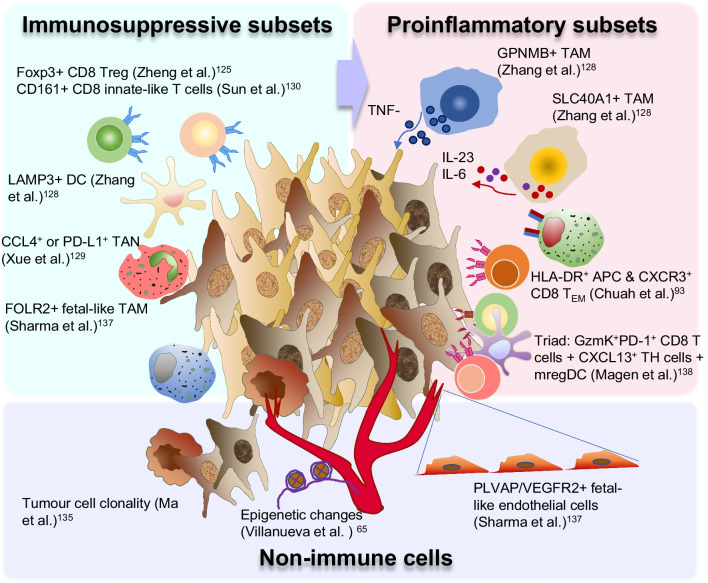
Multi-omics analyses of complex dynamics within the TME of HCC. Several immune subsets, which are either immunosuppressive, pro-inflammatory, or cytotoxic, as observed in multi-omics analyses of HCC. Non-immune cells such as the endothelial and tumor cells also play an key role in the TME of HCC. Treg, regulatory T cells; DC, dendritic cells; TAN, tumor-associated neutrophils; TAM, tumor-associated macrophages; APC, antigen-presenting cells; T_EM_, T-effector memory cells

SC-omics analysis has not only facilitated the characterization of the diverse immune cell subsets within the TME but also substantially contributed to the understanding of immune profiles in different disease states and biomarker discovery for immunotherapy in HCC. In an early SC-transcriptomic study, Zheng et al. [125] analyzed the landscape of T cells in individually sorted CD4^+^ and CD8^+^ T cells from TME, non-TME and peripheral blood of patients with HCC. They comprehensively examined various T-cell populations and identified *LAYN* as the key gene associated with the suppressive function of Treg cells and exhausted CD8 T cells within the TME. In addition to Treg and exhausted CD8 + T cells, they discovered a unique TME-specific *CD8*^+^*FOXP3*^+^ regulatory-like cell population, confirmed by multi-color immunohistochemistry (Fig. 2). They indicated that this Foxp3^+^CD8^+^ Treg cell subset was characterized by the expression of typical Treg genes, including *FOXP3*, *CTLA4*, *TNFRSF9*, and *TNFRSF18*, and cytolytic-related genes, including *PRF1*, *GZMA*, and *NKG7* [125]. Earlier and subsequent studies on Foxp3^+^CD8^+^ Treg cells have suggested an immunosuppressive phenotype [126, 127]. Zhang et al. [128] used a combination of two single-cell RNA sequencing technologies (10 × Genomics and SMART-seq2) to comprehensively analyze the CD45^+^ immune landscapes of five compartments (tumor, adjacent liver, hepatic lymph node, blood, and ascites) from 16 treatment-naive patients with HCC. Focusing primarily on the role of DCs and TAMs in regulating the functions of lymphocytes in the TME of HCC, the authors examined the key roles of the LAMP3^+^ DCs and GPNMB- or SLC40A1-expressing TAMs (Fig. 2) [128]. They reported that the LAMP3^+^ DCs were more likely associated with T-cell dysfunction. In addition, GPNMB^+^ TAMs promoted TNF-α production, whereas SLC40A1^+^ TAMs promoted pro-inflammatory cytokines such as IL-23 and IL-6 but suppressed IL1b production (Fig. 2). The latest addition to this series of single-cell RNA sequencing studies from the same group focused on tumor-infiltrating neutrophils (TANs). They found that the CCL4^+^ and PD-L1^+^ TANs were both immunosuppressive and associated with poor prognosis in patients with HCC (Fig. 2) [129]. This series of single-cell analyses of the TME of HCC has unveiled the complex composition and dynamic interaction of tumor-infiltrating immune cells, thereby providing a valuable resource for understanding and developing strategies aimed at modifying the TME to enhance antitumor immunity.

In addition to the immunophenotyping of the TME of HCC, SC-omics analysis has played a key role in elucidating various immune profiles across different disease states. Nguyen et al. [158] reported distinct immune landscapes with HCC progression, with the peak of immune evasion observed at the intermediate stage, characterized by accumulation of exhausted CD8^+^ T cells and Treg cells. Sun et al. [130] reported an increase in DCs with decreasing antigen-presentation capability and reduced Treg cells in early-relapse HCC cases. They also discovered a unique CD8^+^ T-cell population enriched in early-relapsed HCC, which expressed KLRB1 (CD161) and displayed an innate-like, low cytotoxic and clonal expansion phenotype (Fig. 2). These two studies have indicated that immune evasion is a dynamic process that occurs at different time points along tumor progression and relapse, indicating that anti-CD161 may serve as a potential novel checkpoint target, particularly for relapsed HCC [131].

Multi-omics analysis has achieved great progress in biomarker discovery for therapeutic response [132–134]. Ma et al. [135, 136] examined the clonal evolution of tumor cells and their interaction with immune cells in patients with HCC and cholangiocarcinoma who received immunotherapy (Fig. 2). Sharma et al. [137] reported a similarity between the immune modulation of fetal liver and the TME of HCC and discovered that VEGF and NOTCH signalling play a functional role in maintaining immune-suppressive onco-fetal reprogramming (Fig. 2). This study provides valuable insights into the potential mechanisms and targets of anti-VEGF therapy in HCC. Other studies have indicated that the interaction between effector T cells and DCs [93, 138], macrophages, and cancer-associated fibroblasts [139] regulate immunotherapeutic response. In a study based primarily on the peripheral blood SC transcriptomic analysis, CXCR3^+^ effector memory CD8^+^ T cells and HLA-DR^+^ APCs were identified as two key potentially interacting immune cells linked to distinct clinical fates of either response or immune-related adverse effects (irAEs) in patients with HCC who underwent anti-PD-1 therapy (Fig. 2) [64]. Subsequently, a therapeutic strategy was designed to uncouple response and irAEs for optimal therapeutic outcome [64]. In a more recent study, scRNA-seq and spatial transcriptomic analyses were used to identify a triadic interaction among granzyme K + PD-1 + effector-like CD8 + T cells, CXCL13 + CH25H + IL-21 + PD-1 + CD4 + T helper cells, and LAMP3 + mature DCs enriched in immunoregulatory molecules (mregDC), which are linked to therapeutic response in HCC patients treated with neoadjuvant anti-PD-1 ICIs [138].

In summary, multi-omics studies have offered a comprehensive and multi-dimensional understanding of the TME, thereby laying the foundation for the discovery of novel therapeutic targets for next-generation immunotherapy [132, 140].

### Development in multi-omics driven therapeutic design for HCC

At present, the most popular approach in clinical trials is to combine anti-PD1/ anti-PDL1 ICIs with another ICI agent, such as anti-TIM3 [141], anti-LAG3 [142], or anti-TIGIT [143], to enhance the re-invigorating effects of antiPD1/ anti-PDL1 ICIs on exhausted CD8 T cells [144]. Chiu et al. [145] compared human HCC to adjacent non-tumor liver tissues and observed an increase in *PVRL1*, which stabilizes cell surface poliovirus receptor (PVR) that interacts with TIGIT. They reported that TIGIT inhibition or genetic ablation of *PVRL1* increased ratio of cytotoxic CD8 + T cells to Treg cells in murine liver cancer models and sensitized the mice to anti-PD1 therapy (Table 4). Wei et al. [146] identified a signaling pathway linking protein kinase C alpha (PKCα), the transcription factor ZPF64, and colony-stimulating factor-1 (CSF-1), which plays a key role in polarization of TAMs towards an immunosuppressive M2 phenotype in the TME of HCC and resistance to anti-PD-1 therapy. They also discovered potent antitumoral activity in preclinical models when inhibitors targeting PKCα (Gö6976) or CSF1 (BLZ945) were combined with anti-PD-1 therapy, suggesting new options for reversing resistance to anti-PD-1 therapy (Table 4).

**Table 4:** Proposed combination therapies from multi-omics studies

Proposed therapy	Analysis tools involved	Key findings	References
Anti-Tigit + anti-PD-1	CyTOF, Immunohistochemistry (IHC)/ Immunofluorescence and flow cytometry	• Combination of anti-TIGIT plus anti-PD1 reduced tumor burden and prolonged survival in HCC model • Combination therapy increased the ratio of cytotoxic CD8 T cells to regulatory T cells in tumor	Chiu et al. Gastroenterology, 2020 [145]
Anti-TNFR2 + anti-PD-1	CyTOF, scRNA seq, multiplex Immunofluorescence and flow cytometry	• TNFR2 is specific biomarker related to response to anti-PD-1 ICI • Anti-TNFR2 + anti-PD-1 provides uncoupled effect with enhanced response without the increase in irAEs	Chuah et al. J Hepatol, 2022 [93]
Metformin + anti-PD-1	Intravital imaging, transcriptomic analysis and flow cytometry	• NASH-dependent impairment of hepatic CD8^+^ T-cell metabolism leading to impaired response to anti-PD-1 in mice with NASH-HCC • The use of Metformin could reverse such impairment and improve the response to anti-PD-1 therapy	Wabitsch et al. J Hepatol 2022 [105]
AZD5069 (CXCR2 small molecule inhibitor) + anti-PD1	Imaging mass cytometry, RNA-seq and flow cytometry	• CXCR2-inhibition in NASH-HCC model reprogrammed the TME and enhances response to ICI • CXCR2 inhibition reprogrammed tumour-associated neutrophils (TANs) to a cytotoxic and anti-tumoral phenotypes • CXCR2 inhibition increases intratumoral XCR1 + dendritic cells and cytotoxic CD8^+^ T cell	Leslie et al. Gut, 2022 [147]
Gö6976 (PKCα inhibitor) or BLZ945 (targeting CSF1R) + anti-PD-1	CyTOF, multiplex immunofluorescence and RNA-sequencing	• Phosphorylation of ZFP64 promotes transcriptional activation of CSF1 and immunosuppressive M2 macrophage polarization • PKCα was identified as the upstream kinase for ZPF64 phosphorylation and hence by targeting PKCα (Gö6976) or CSF1 (BLZ945) in combination with anti-PD-1 therapy demonstrated enhanced anti-tumour activity	Wei et al. J Hepatol 2022 [146]
Cabozantinib (multikinase inhibitor) + anti-PD-1	Flow cytometry, IHC, transcriptome and cytokine profiling as well as multiplex immunofluorescence	• Esteban-Fabro et al. reported a neutrophils-medicated anti-tumour immune response enhanced by the combination of cabozantinib and anti-PD-1 • Ou et al. reported the main anti-tumour effect of the combination via the suppression of MDSCs	Esteban-Fabro et al. Clin Cancer Res 2022 [153]. Ou et al. Ann Oncol 2022 [155]

Because the efficacy and adverse events of ICI therapy are both immune-related, uncoupling these events to enhance efficacy without aggravating adverse events will greatly improve the therapeutic index of new combination regimens. Chuah et al. [93] identified the interaction between CXCR3^+^ effector memory CD8^+^ T cells and HLA-DR^+^ APCs as a key mechanism determining response versus irAEs in patients with HCC treated with anti-PD-1 ICI. They identified TNFR2 as a key biomarker specifically linked to clinical response but not irAEs, and demonstrated enhanced therapeutic response without increased irAEs in preclinical models by combination of anti-TNFR2 and anti-PD-1. They also discovered that TNFR2 was specifically enriched in Treg cells within the TME of HCC, indicating a potential tumor Treg-specific target (Fig. 2). Overall, these findings may facilitate the development of therapeutic strategies aimed at uncoupling therapeutic response and irAEs to optimize therapeutic outcome (Table 4).

Multiple studies have examined the mechanisms underlying the immune-suppressive TME associated with NASH- or MASLD-related HCC [147]. According to preclinical models, the NASH microenvironment may induce CD8 T-cell subpopulations that caused liver damage [106] or even promote HCC development [104]. Wabitsch et al. [105] reported that the metabolic reprogramming of hepatic CD8^+^ T cells resulted in impaired motility and resistance to anti-PD-1 therapy in murine NASH-HCC models. They indicated that this dysfunctional CD8^+^ T-cell phenotype was reversed by metformin treatment (Table 4). Many studies have extensively examined the cancer-preventing effects of metformin, and numerous mechanisms have been proposed [148–151]. Leslie et al. [147] identified TANs, which over-expressed the neutrophil receptor CXCR2, as key factors underlying the inferior efficacy of anti-PD-1 therapy in NASH-related HCC. They reported that combining anti-PD-1 with AZD5069, a CXCR2 inhibitor, led to the reprogramming of TANs to a more proliferative and inflammatory phenotype, increased intra-tumoral XCR1^+^ DCs and CD8^+^ T-cell infiltration, and enhanced anti-tumor response in NASH-HCC models (Table 4). In summary, multi-omics approaches can be used to clarify the immune modulatory mechanisms of the underlying liver diseases and to identify novel therapeutic targets in the TME of HCC.

Research into the immunomodulatory effects of MKIs should not be limited to their anti-angiogenic properties. Lenvatinib may inhibit the PKCα/ZFP64/CSF1 [146] and transforming growth factor-β signaling pathways in the TME of HCC (Table 4) [152]. Cabozantinib may also increase neutrophil chemotaxis, induce infiltration of TANs [153] with a more cytotoxic N1 phenotype [154], and reduce intra-tumoral myeloid-derived suppressor cells (MDSCs) (Table 4) [155]. Although these immunomodulatory mechanisms of MKIs may enhance effector T-cell infiltration and response to anti-PD1 therapy in preclinical HCC models, the lack of additional survival benefits provided by combination therapy in randomized clinical trials indicates that additional comprehensive mechanistic studies are required to determine whether and how these mechanisms enhance antitumor immunity in clinical settings.

Epigenetic regulation plays a key role in modulating antitumor immune response through both innate and adaptive immunity. Epigenetic modifiers such as de-methylating agents, HDAC inhibitors, and EZH2 inhibitors, can activate NK cells and macrophages, reverse CD8 T-cell exhaustion, and suppress Treg-mediated immune suppression [67, 70]. Among all types of epigenetic modifiers, HDAC inhibitors are the most widely evaluated in pre-clinical models of HCC [120, 156, 157]. Nevertheless, identifying the most relevant cellular and molecular targets of HDAC inhibitors is a challenging task. Therefore, conducting multi-omics analyses at the single-cell level can aid in elucidating the evolution of immune cells, dissecting the intra-tumor heterogeneity, and identifying rare but functionally essential cell populations [21].

### Future perspectives

In this review, we highlighted the potential of advanced technologies in addressing the limitations in the current process of drug development and biomarkers discovery for HCC. These technologies can provide a more comprehensive understanding of the heterogeneity and complexity of the TME, which can consequently clarify the mechanisms underlying various treatment options for HCC. As creators, translational researchers should be aware of the most recent advance of the novel technologies to rapidly and accurately identify new biomarkers and treatment options.

## Conclusion

Integrating advanced multi-omics technologies into clinical trials, from early proof-of-concept trials involving novel combination strategies to pivotal trials versus the current standard of care, requires close collaboration between translational researchers and clinical trial specialists to push the frontiers of HCC treatment toward a definitive cure.

## Data Availability

Not applicable.
